# Morphological Variability Identification of *Harumanis* Mango (*Mangifera indica* L.) Harvested from Different Location and Tree Age

**DOI:** 10.21315/tlsr2020.31.2.6

**Published:** 2020-08-06

**Authors:** Siti Nur Arina Yusuf, Ahmad Mukhlis Abdul Rahman, Zarina Zakaria, Vijay Kumar Subbiah, Maz Jamilah Masnan, Zakaria Wahab

**Affiliations:** 1 Department of Chemical Engineering Technology, Faculty of Engineering Technology, Universiti Malaysia Perlis, Sungai Chuchuh, 02100 Padang Besar, Perlis, Malaysia; 2 Biotechnology Research Institute, Universiti Malaysia Sabah, Jalan UMS, 88400 Kota Kinabalu, Sabah, Malaysia; 3 Institute of Engineering Mathematics, Universiti Malaysia Perlis, Kampus Pauh Putra, 02600 Arau, Perlis, Malaysia; 4 Department of Mechanical Engineering Technology, Faculty of Engineering Technology, Universiti Malaysia Perlis, Sungai Chuchuh, 02100 Padang Besar, Perlis, Malaysia

**Keywords:** *Harumanis*, Geographical Region, Morphological Variation, *Harumanis*, Kawasan Geografi, Kepelbagaian Morfologi

## Abstract

*Harumanis* is one of the main signatures of Perlis with regards to its delightful taste, pleasant aroma and expensive price. *Harumanis* authenticity and productivity had become the remarks among the farmers, entrepreneurs, consumers and plant breeders due to the existence of morphological characteristics variation among the fruits and high production cost. Assessment of *Harumanis* morphological characteristics of natural population and different tree ages may represent a possible source of important characteristics for development and breeding purposes of *Harumanis*. The aim of this study is to evaluate the morphological variation of *Harumanis* collected from different location in Perlis and tree age. A total of 150 *Harumanis* fruits from 50 trees with three different stages of development (young, middle-aged and old) were characterised using 11 traits; 10 quantitative and one qualitative morphological trait. The ANOVA analyses in combination with Dunn’s pairwise and Kruskal-Wallis multiple comparison test able to point out the existence of environmental factor and age influence towards the significant different of identified morphological traits except for Total Soluble Solid (TSS) and pulp percentage. Five clusters of 50 *Harumanis* accessions reflect a grouping pattern which not according to neither geographical region nor age. The result of Principal Component Analysis (PCA) using the first two principal components (PCs) provided a good approximation of the data explaining 84.09% of the total variance which majorly contributed by parameters of weight, fruit dimensional characteristics, peel percentage and hue angle, *h*. Preliminary screening of important morphological characteristics which contribute to the phenotypic diversity of *Harumanis* is successfully achieved. The findings can be employed by the plant breeders and farmers for the establishment of standard grading of *Harumanis* and advancement of breeding crop of *Harumanis* in future.

## Abstract (ms)

*Harumanis* ialah salah satu tanda Perlis kerana rasanya yang menarik, aroma yang wangi dan harganya yang mahal. Keaslian *Harumanis* dan produktivitinya telah menjadi tanda dalam kalangan petani, peniaga, pembeli dan pembiak baka tumbuhan kerana adanya kepelbagaian ciri morfologi antara buah dan kos pengeluaran yang tinggi. Ciri-ciri morfologi *Harumanis* dinilai dari segi populasi semula jadi dan usia pokok yang berbeza yang berkemungkinan menjadi ciri penting untuk perkembangan dan pembiakan *Harumanis*. Tujuan kajian ini adalah untuk menilai kepelbagaian morfologi *Harumanis* yang dikutip daripada beberapa lokasi berbeza di Perlis dan juga berdasarkan umur pokok. Sebanyak 150 buah *Harumanis* daripada 50 buah pokok dengan tiga peringkat perkembangan (muda, pertengahan dan tua) dan dibezakan menggunakan 11 trait; 10 kuantitatif dan 1 kualitatif. Analisis ANOVA digabungkan dengan ujian perbandingan pelbagai pasangan Dunn dan Kruskal-Wallis yang dapat menunjukkan keberadaan faktor persekitaran dan pengaruh usia terhadap perbezaan sifat morfologi yang dikenal pasti, kecuali jumlah peratusan larut (TSS) dan peratusan pulpa. Lima kelompok 50 aksesi *Harumanis* menunjukkan corak pengelompokan yang tidak sesuai dengan wilayah geografi atau usia pokok. Hasil analisis komponen utama (PCA) menggunakan dua komponen utama (PC) memberikan penghampiran data yang baik yang menerangkan 84.09% dari jumlah varians yang sebahagian besarnya disumbangkan oleh parameter berat, ciri dimensi buah, peratusan kulit dan sudut rona, *h*. Pemeriksaan awal ciri-ciri morfologi penting yang menyumbang kepada kepelbagaian fenotipik *Harumanis* berjaya dicapai. Penemuan ini dapat digunakan oleh pembiak baka tumbuhan dan petani untuk menetapkan penilaian standard *Harumanis* dan kemajuan pembiakan tanaman *Harumanis* di masa depan.

**Highlights**

- Fifty accessions of *Harumanis* harvested from different location and tree age were evaluated based on their morphological variation.
- The result of Principal Component Analysis (PCA) provided a good approximation of the data which majorly contributed by parameters of weight, fruit dimensional characteristics, peel percentage and hue angle, *h*.
- Preliminary screening of important morphological characteristics which contribute to the phenotypic diversity of *Harumanis* is successfully achieved.

## INTRODUCTION

*Harumanis* is a fruit belongs to *Mangifera* genus which also known as MA 128 (Jabatan Pertanian Malaysia 1995) and has been registered on 28 May 1971 by the Department of Agriculture, Malaysia (Aziah 2006). This variety is known for its pleasant taste, texture and fragrance among consumers and is popular both in the local and international markets (Jha *et al*. 2013). These sensory parameters vary with individual mango and person involved in testing. There is little or no information available on the instrumental estimation of these sensory attributes. The present study, therefore, was conducted to correlate sensory and instrumental textural attributes to explore the possibility of predicting them for seven major cultivars of mango influenced by harvesting dates and ripening period. The fruit shape of *Harumanis* is oblong with prominent beak, while the skin shading is green and usually will turn to yellowish green when ripe. The fruit size varies, ranging from 300 to 650 grams, has a 16° to 17° Brix value and the flesh colour is usually orange and sweet (Sani *et al*. 2018).

Despite its appealing qualities, *Harumanis* is facing a problem which related to the production inconsistency due to phenotype variation which caused the flesh colour and sizes of the fruits to be varied although they are harvested from the same cultivation area. This situation is worsening as some other mangoes like *Sala* and *Tong Dam* look almost similar and leads to confusion about clear identification of this exceptional mango. The current grading system of *Harumanis* is via visual morphological inspection and the technique is also used for various mangoes including *Sala, Chokanan* and *Maha* (Chee 2009; Jusoh 2017). This has been a major drawback among consumers since they can misjudge other mangoes as *Harumanis* and buy them at expensive price (Bakhtiar 2011). Eventually, such concerns will give a negative consequences to the markets, especially in term of revenue and prominence of Perlis as the main producer of *Harumanis* in Malaysia (Jusoh 2017).

Morphological marker has been classified as one of the factors that play a major role in plant breeding and can be applied to pre-determine the agronomic traits of interest. Identification of variation and traits of interest, followed by its incorporation into germplasm are the influential components of any crop advancement for plant breeding programme. Such variation can be obtained from either crossing two distinctive parental genotypes or choosing existing variations from the highly available germplasm from natural populations (Rajeev *et al*. 2009). So far, very few reports have been published with regards to the evaluation of genetic and phenotypic diversity of *Harumanis* in this country with limited number of morphological traits being studied (Ariffin *et al*. 2015; Rahman *et al*. 2018; Sani *et al*. 2018; Yusuf *et al*. 2018a; 2018b). The systematic study on morphological variation of *Harumanis* fruit samples collected from various location and tree age in Perlis has never been reported previously.

Morphological marker by performing trait identification may give several advantages such as simple, rapid and cost-effective assays, even from herbarium specimens to other dead tissues (Begum *et al*. 2013). It has been proven useful for identifying accessions in many fruit trees such as cherimoya (*Annona cherimola* Mill) (Andres-Agustina *et al*. 2006), cherry (*Prunus incana* Pall) (Aliyoun-Nazari *et al*. 2012), cashew (*Anacardium occidentale* L.) (Chabi *et al*. 2015), peach (*Prunus persica* L.) (Chalak *et al*. 2013), cherry (*Prunus cerasus*) (Ganopoulos *et al*. 2016), durian (*Durio zibethinus*) (Handayani & Ismadi 2018), and Amrapali mango (Meena & Asrey 2018). Many of the characteristics analysed have potential economic interests, especially those related to mango such as fruit weight (Faridah *et al*. 2010), total soluble solid (Mulando *et al*. 2001) and colour (Ornelas-Paz *et al*. 2008).

The presence of intra-varietal among the fruits is usually caused by various factors. It is probably due to phenotypic variation caused by spontaneously arisen genetic polymorphisms from their relatives and ancestors that are maintained by nature, adaptation to different natural environment and human preferences (Kliebenstein 2009). Furthermore, the development factor such as type of rootstock and scion may also cause the variation (Vieira *et al.* 2007). Therefore, having abundant choices of parents from different location for population development mapping and extending the mapping with the fruits’ quantitative and qualitative characteristics by using morphological marker will be an effective method for selection of superior progenies. The parent crops with traits of interest such as higher yield, improved nutritional quality and appropriate colour or fragrance preferred by consumers can be singled out and be used as the preferred crop for breeding purposes.

Other than environmental effect, tree age also affects the nutritive properties, storage and physiology of harvested fruits (Meena & Asrey 2018). The tree age factor affects the pomological parameters of the olives and the physiochemical characteristics of the virgin olive oil (Bouchaala *et al*. 2014). Young tree of orange was reported to produce low quality of fruits compared to the older trees (Hearn 1993). The tree age also reported to affect the fruit yields and physical characteristics of pummelo (Nakorn & Chalumpak 2016) and apple fruits (Arshad *et al*. 2014). Ozeker (2000) reported that a 20-years-old “Marsh seedless” tree produced heavier grapefruits with higher juice content, soluble solids and acidity compared to fruits from a 34 years old tree.

Overall assessment of the important and highly potential mango traits needs to be analysed to be further utilised for *Harumanis* crop breeding improvement. Thus, this study is the first attempt for the assessment of *Harumanis* variability based on the morphological traits from different location in Perlis and tree age using morphological methods by performing statistical analyses by ANOVA and Kruskal-Wallis test in combination with Dunn’s pairwise multiple comparison test, clustering analysis by Ward’s method and squared Euclidean distances with the combination of PCA. Further identification of morphological key descriptors will be helpful in assisting the selection of desirable individuals with high commercial morphological values.

## MATERIALS AND METHODS

### Study Area

A total of five different orchards around Perlis were selected for the study. The orchard selection was based on several criteria such as dispersion of locations which covering north, south and east of the state, different tree ages which categorised as young (5 to 10 years old), middle-aged (15 to 20 years old) and old (33 to 35 years old) (Khalid *et al*. 2017; Meena & Asrey 2018) and farmers who are certified with MyGAP (Malaysian Good Agricultural Practices). The location of each of the sampled trees was recorded using a handheld global positioning system (GPS) along with location information of the surveyed trees (Table 1). A random sample of 50 *Harumanis* mango trees were selected as stated in Table 1 for characterisation purpose.

### Plant Materials

Fruit sampling and morphological characterisation were conducted during *Harumanis* harvesting season from March till June 2017. Triplicate of mangoes per accessions were hand-harvested as they reached maturity (13 weeks after flowering) and further undergo a post-harvest process prior analysis (Khidir & El Tayeb 2014).

### Morphological Descriptors and Statistical Data Analysis

The morphological diversity observation within the studied population was determined by a total number of 10 quantitative and one qualitative scored morphological trait as indicated. The 10 quantitative traits analysed are fruit weight, length, width, thickness, peel firmness, flesh firmness, total soluble solid (TSS), peel percentage (% (w/w) of total fruit), pulp percentage (% (w/w) of total fruit) and stone percentage (% (w/w) of total fruit) while hue angle is the only qualitative trait. TSS was obtained by measuring the top, middle and bottom part of the pulp juice of the equatorial region of fruit using an Atago Digital Refractometer.

The TSS was measured at different positions of the fruits (top, middle and bottom) as sugar levels often vary within the fruit, being higher at the stem-end and lower at the calyx-end. Thus, the value of the TSS is recorded as a mean from each position’s reading in each sampled fruit (Jha *et al*. 2010). For flesh colour, the internal colour reading was measured using a portable colorimeter (CR-400 Minolta) at the central region of both equatorial regions of the mango (L*, a* and b*), in which L* value indicates the lightness [black [L* = 0] and [L* = 100]], a* value indicates redness-greenness (red [a* = 100] and green [a* = (−100)]) and b* indicates yellowness-blueness (yellow [b* = 100] and blue [b* = (−100)]). Hue is derived from a* and b* using the following equation:

$$\text{Hue angle }[\text{h}=\text{arc tan }(b^{*}/a^{*})]$$(
Varakumar *et al*. 2011)

The colour coordinate was able to show the variation between the basic colours. Minimum, maximum, mean and coefficient of variation (CV) were computed for each trait by using Excel 2013. The CV was determined as the variability indicator. The CV percentage was determined as the variability index (Markovski & Velkoska-Markovska 2015). The ANOVA, clustering, dendrogram and PCA were performed using SPSS and IBM 19.0 and Minitab 16. Comparison of traits heterogeneity among the accessions across location and tree ages (Meena & Asrey 2018) were performed by ANOVA followed by Tukey post hoc test for traits across categories (tree age: length, thickness and peel firmness and location: weight, length, width, thickness and peel firmness). Kruskal-Wallis test followed by Dunn-Bonferroni pairwise comparisons test were preferred for the remaining traits across both categories since the distributions are not symmetric (Bing *et al*. 2018).

$$T=(N-1)\frac{\sum_{i=1}^{k}n_{i}{\left(\bar{r_{i}}-\bar{r}\right)}^{2}}{\sum_{i=1}^{k}\sum_{j=1}^{n_{i}}{\left(r_{ij}-\bar{r}\right)}^{2}}$$

Where *k* is the number of groups, *n_i_* is the sample size of sample *I*, *N* is the total number of all the observations across *k* groups, *r_ij_* is the rank (among all the observations) of observation *j* from group *i.*

These tests were performed to test the hypotheses of the distribution of morphological traits mean (*H_0_: μ_U_ = μ_E_*) against the two-tailed alternative (*H_1_: μ_U_ ≠ μ_E_*) for ANOVA if normality is assumed or if normality is not assumed median (*H_0_: median_U_ = median_E_* against *H_1_: median_U_ ≠ median_E_*) for Kruskal-Wallis test if the distribution of the accessions are not same across both categories of age and place depending on the result of a Shapiro-Wilk normality test (Dunn 2013).

Correlations between traits were computed using Spearman’s correlation coefficient and *p*-value correlation matrix. The distances between the traits were then computed using the squared Euclidean norm based on similarity levels, using standardised data. A hierarchical cluster analysis was conducted in order to group the samples according to their morphological similarities. The patterns of the main variations and the most discriminant traits were analysed using PCA. Clustering was performed after *Z*-score standardisation of qualitative and quantitative variables using squared Euclidean distances and the Ward method (1963) by using only the selected key descriptors which present significant different across categories (location and place) and have high correlation between each other (Abdi & William 2010).

## RESULTS

Results of this study were divided into sections which comprised of fruit characterisation by performing descriptive statistics, analyses of variance, pairwise comparison, PCA and clustering analysis.

### Fruit Characterisation

Descriptive statistics i.e., mean, range, variance, standard deviation and coefficient variation results for 11 morphological traits are presented in Table 2. In fruit characterisation, high variations of few morphological descriptors were found among the 50 *Harumanis* accessions. The fruit weight varied extensively from 263.00 g to 670.33 g. Fruit size varied among the samples from 10.00 cm to 14.31 cm in length, 6.07 cm to 8.45 cm in diameter and 5.63 cm to 7.69 cm in thickness. Peel firmness ranged between 3.05 N s^−1^ to 7.24 N s^−1^, flesh firmness ranged between 0.70 to 2.93 N s^−1^ and the Brix values of the fruits ranged from 14.6° to 18.23°. The hue angle varied from 68.09° to 81.09°. For mass composition, the highest peel, pulp and stone composition are 18%, 74% and 19%, respectively. Whereas, the lowest peel, pulp and stone percentage are 10%, 53%, and 9%, respectively. The hue angle varied from 68.09° to 81.09° as indicated in Table 2. Any morphological trait showing CVs greater than 20.00% indicates a high variability between the genotypes (Norouzi *et al*. 2017). The highest variations identified are flesh firmness (34.47%) and fruit weight (20.62%) followed by peel firmness (18.16%), peel percentage (14.94%) and stone percentage (14.23%). These high CV values indicate the existence of high range of selection for those traits (Mohammadi & Prasanna 2003). The lowest CV value is shown by the hue angle with a CV of 4.31%. The morphological traits with low variation are more homogeneous and can be repeated between accessions, and therefore may be considered as a stable feature (Khadivi 2018).

### ANOVA and Pairwise Comparison

All of the data set were analysed using Kruskal-Wallis test due to the violation of normality assumption, except for fruit length, width, thickness and peel firmness were analysed using ANOVA across locations. Out of 11 morphological traits, nine morphological traits which are fruit weight, length, width, thickness, peel firmness, pulp firmness, peel percentage, stone percentage and hue angle showed a significant difference (*p* < 0.05) across different geographical region and tree ages from ANOVA and Kruskal-Wallis test. For location, there is a statistically significant effect of location on fruit weight, length, width, thickness and peel firmness at the (*p* < 0.05) level for the five locations [*F* (4, 45) = 6.516, 8.665, 8.275, 13.811 and 10.487, *p* = 0.000] (Tables 3 and 5).

**Figure 1:**
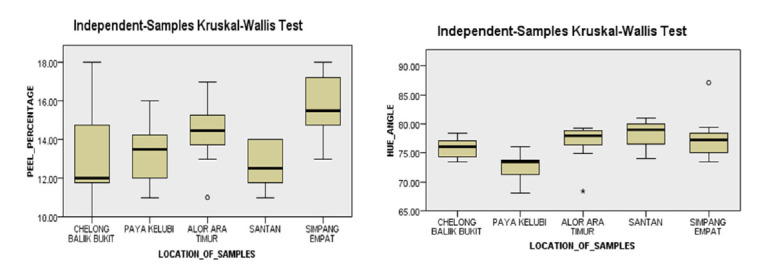
Box plots of values extracted from peel percentage for five locations. The thick horizontal line inside each box represents the median; the upper and lower borders of the box are the upper and lower quartile; the lower and upper whisker are represented by the error bars. Small circles represents outliers in the data and asterisks indicated significant differences from Dunn’s post hoc test using Bonferroni corrections.

The results in a probability value which below the α level (*p* < 0.05) resulted the rejection of null hypotheses which means that there is presence of differences in the parameters across the categories (location and tree age), Tukey post hoc test in Table 4 and post-hoc Dunn’s test in Fig. 1 with Bonferroni correction were performed to determine which means or median amongst a set of means or median differ from the rest (Bland & Altman 1995). Notice in Table 4, post hoc comparisons using the Tukey HSD test indicated that the mean score for *Harumanis* Santan (M = 151.567, SD = 33.319) weight is statistically significant different in positive effect at the *p* < 0.05 level when compared with *Harumanis* Simpang Empat. For length, the means difference was statistically significant in negative effect when *Harumanis* Simpang Empat became the (I). The length difference was slightly lower compared to *Harumanis* Chelong Balik Bukit, Paya Kelubi and Santan. Another fruit dimensional characteristic i.e. fruit width showed statistically significant difference in negative effect again between *Harumanis* Simpang Empat when compared to *Harumanis* Chelong Balik Bukit, Paya Kelubi and Santan. Nevertheless, only the comparison between *Harumanis* Simpang Empat and *Harumanis* Santan presented a significant difference at *p-*value of less than 0.05. *Harumanis* Paya Kelubi showed a positive mean difference of length trait when compared to *Harumanis* Alor Ara Timur with a mean score of (M = 0.928, SD = 0.313) indicated a greater length of *Harumanis* Paya Kelubi than *Harumanis* Alor Ara Timur. Meanwhile, for thickness, this test revealed that the thickness of *Harumanis* Simpang Empat is lower compared to thickness of *Harumanis* Chelong Balik Bukit and *Harumanis* Santan. Both showed statistically significant difference which implied by the positive mean difference of (M = 0.904, SD = 0.1524) when compared with *Harumanis* Chelong Balik Bukit, while the mean score when compared with *Harumanis* Santan was (M = 1.032, SD = 0.152) as these both locations became the (I). *Harumanis* Alor Ara Timur scored a negative mean difference (M = −0.488, SD = 0.152) of thickness when compared to *Harumanis* Santan with a significant difference of *p* < 0.05.

The results of Kruskal-Wallis rank sum test determined whether the morphological traits of peel percentage and hue angle are varied across different locations. According to Table 5, the *Harumanis* accessions peel percentage median of the five locations were 14.50%, 12.00%, 13.50%, 12.50% and 15.50%. The average rank exposed that *Harumanis* Simpang Empat group median was the highest, while *Harumanis* Chelong Balik Bukit group median was the lowest. This relationship can be clearly seen when performing the Dunn-Bonferroni pairwise comparison test by comparing the boxplot as illustrated in Fig. 1. *Harumanis* Simpang Empat attained the highest peel percentage because the boxplot displayed the highest median and *Harumanis* Chelong Balik Bukit attained the lowest peel percentage as the median was the lowest when compared to the peel percentage attained by *Harumanis* accessions from other locations. For hue angle, the Kruskal-Wallis test presented a significant result which indicated by the *p*-values less than 0.05 (Table 5). By referring to the hue angle box plot, *Harumanis* accessions of Paya Kelubi acquired the most dense flesh colour due to the lowest median among the groups and the median was near the upper quartile (Fig. 1).

Kruskal-Wallis test followed by Dunn-Bonferroni pairwise comparisons test was used in multiple comparisons to analyse the variation pattern of morphological traits of *Harumanis* across different age categories of young (5 to 10 years old), middle-aged (15 to 20 years old) and relatively old (33 to 35 years old) as the result violated the normality assumption as indicated by Shapiro Wilk test. Out of 11 morphological traits, seven traits presented significant differences with the *p*-value less of than 0.05 as indicated in Table 6. These results enabled the rejection of null hypotheses of equal distribution of parameters across tree age.

**Figure 2:**
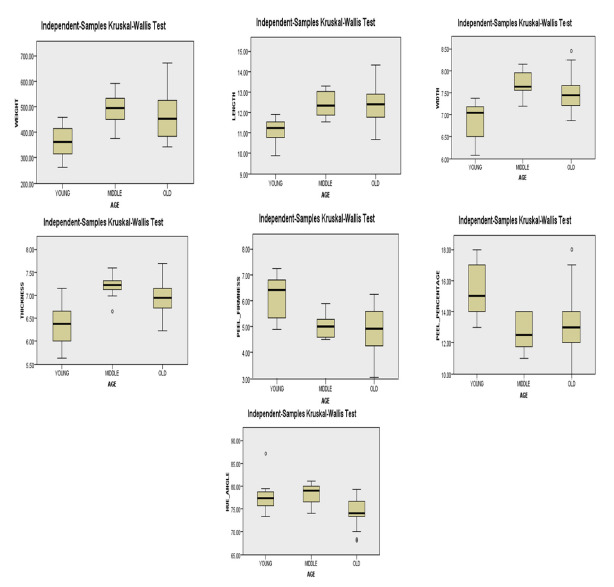
Box plots of values extracted from peel percentage for five locations. The thick horizontal line inside each box represents the median; the upper and lower borders of the box are the upper and lower quartile; the lower and upper whisker are represented by the error bars. Small circles represents outliers in the data and asterisks indicated significant differences from Dunn’s post hoc test using Bonferroni corrections.

According to Table 6, the fruit weight average rank showed that the *Harumanis* accessions from middle-age (15 to 20 years old) trees differed the most amongst all observations. The highest *Z* value was attained by the *Harumanis* accessions from middle-age (15 to 20 years old) trees, which is 1.44. The positive *Z* value indicated that the *Harumanis* accessions of middle-age (15 to 20 years old) trees average rank were greater than the overall average rank. Table 6 indicated that the *Harumanis* accession from old trees presented lower median (M = 451.8 g) compared to middle-age (15 to 20 years old) trees and presented the most diverse fruit weight as the box plot was comparatively tall. The young (5 to 10 years old) tree of *Harumanis* accession are having smallest size due to lowest median value attained which was 361.5 g. Both young (5 to 10 years old) and middle-age (15 to 20 years old) trees attained similar length for upper and lower whisker (Fig. 2), indicated the variation pattern of the fruit weight is similar in the age categories whilst, the whisker extended longer for old trees (33 to 35 years old) indicated the fruit weight for old tree *Harumanis* accession were varied more for young (5 to 10 years old) and middle-age (15 to 20 years old) tree *Harumanis* accession.

The fruit dimensional characteristics, fruit length, width and thickness showed similar pattern for those parameters as the *Harumanis* accessions from middle-aged (15 to 20 years old) trees presented the highest median and the lowest median was obtained by the *Harumanis* accessions from young trees (5 to 10 years old) (see Table 6 and Fig. 2). In spite of that, the young trees held the tallest box plot comparatively indicated the most diverse width and thickness.

The dimensional characteristic of fruit length exposed a contradiction finding as *Harumanis* fruit from young (5 to 10 years old) trees acquired the lowest median, followed by middle-aged (15 to 20 years old) trees and relatively old (33 to 35 years old) trees. For length, the lowest average rank of 11.8 cm was scored by young (5 to 10 years old) tree *Harumanis* accessions and the negative *Z*-score of −4.77 indicated that the group average rank was less than normal average rank. The box plot (Fig. 2) indicated by this group was comparatively short suggested that the overall accessions had lower differences of length with each other. Meanwhile, both middle-aged (15 to 20 years old) and old (33 to 35 years old) *Harumanis* trees presented not much differences by obtaining median scores of 12.35 cm and 12.41 cm, distinctively. The width box plot for middle-age trees shows uneven size of the sections indicating uneven distribution of *Harumanis* accessions in this group into two different scales (Fig. 2).

The box plot illustrated a longer whisker in negative direction for both young (5 to 10 years old) and middle-age (15 to 20 years old) trees in fruits width denoting distribution with a negative skew, while the old (33 to 35 years old) trees attained longer whisker in positive direction suggesting a distribution with a positive skew. Fig. 2 illustrates a comparatively short box plot for thickness presented by middle-age (15 to 20 years old) trees which suggested a low variety of thickness between the accessions, while both young (5 to 10 years old) and old (33 to 35 years old) trees exposed a comparatively tall box plots indicated the accessions from these age categories are having quite differ thickness among each other.

Peel firmness revealed the highest median of 6.420 N s^−1^ for *Harumanis* accessions from young (5 to 10 years old) trees whereas the old (33 to 35 years old) trees showed the lowest median of 4.916 N s^−1^. The *Harumanis* accessions from middle-age (15 to 20 years old) trees present a slightly higher median than the median of old (33 to 35 years old) trees. These results revealed that the highest peel firmness was acquired by *Harumanis* accessions from young trees (5 to 10 years old). This variation pattern is in line with the peel percentage findings as the young trees (5 to 10 years old) of *Harumanis* accessions are again having the highest median score and average rank (M = 15.00%; Ave Rank = 37.4). Nevertheless, the *Harumanis* from middle-age (15 to 20 years old) and old (33 to 35 years old) trees presented a not much different medians indicated similar peel percentage attained by the *Harumanis* from both categories.

Among the three different age categories, old (33 to 35 years old) trees presented the least median value of hue angle which was 74.50 indicated the most dense flesh colour attained from this group of *Harumanis* accessions. The old (33 to 35 years old) trees obtained a negative *Z*-score of −3.82, suggesting that most of the accessions scored below the average rank (Table 7). Based on the box plots, both *Harumanis* accessions from young (5 to 10 years old) and middle-age (15 to 20 years old) trees illustrated a longer lower whisker which exhibited that most of accessions from these two categories were varied in negative quartile group.

### Correlation Analysis

The bivariate Spearman’s correlation coefficient indicated by Table 7 reveals a significant correlation between fruit dimensional characteristics, peel percentage and stone percentage as indicated in Table 7. Strong positive linear correlation (*p* < 0.05) were found between fruit weight with length (*r_s_* = 0.896), width (*r_s_* = 0.909) and thickness (*r_s_* = 0.896). Meanwhile, negative linear correlation were found between the peel percentage by weight (*r_s_* = −0.468), length (*r_s_* = −0.567), width (*r_s_* = −0.519) and thickness (*r_s_* = −0.519). Likewise, stone percentage also presented a negative linear correlation with weight (*r_s_* = −0.472), length (*r_s_* = −0.572), width (*r_s_* = −0.606) and thickness (*r_s_* = −0.579). There is also a weak significant correlation between fruit brix and hue angle with *r_s_* = −0.300 (*p* < 0.05) as the *r* value was distant from 1.

### Principal Component Analysis (PCA)

A set of six diverse morphological traits were selected from the 11 traits, namely, weight, length, thickness, width, peel percentage and hue angle to be analysed using PCA. Julnes (2012) suggested PCA procedure to be carried out for multivariate continuous data where multicollinearity exist among variables with the absent of outliers. These assumptions have been checked and fulfilled before PCA procedure was implemented. Table 8 illustrates the total variation explained by each PC as well as the variables’ contribution accordingly. However, the eigenvalues were used to select factors that discriminate the best between the entries. As a criterion to extract the main PCs, Kaiser criterion (eigenvalue greater than 1.00) was referred (Khadivi 2018). One significant component with eigenvalue greater than one was obtained that explained 67.72% of the total variation. However, by referring to the loading value, two PCs which summed up to 84.09% were chosen to explain the variation as loading value above 0.53 was considered as significant as mentioned in previous study (Ganopoulos *et al.* 2016). According to Table 8, the first PC has strong positive association with weight, length, width and thickness with total variance explained of 67.7%. The second PC accumulated 16.4% of variability and largely associated with hue angle and peel percentage.

**Figure 3:**
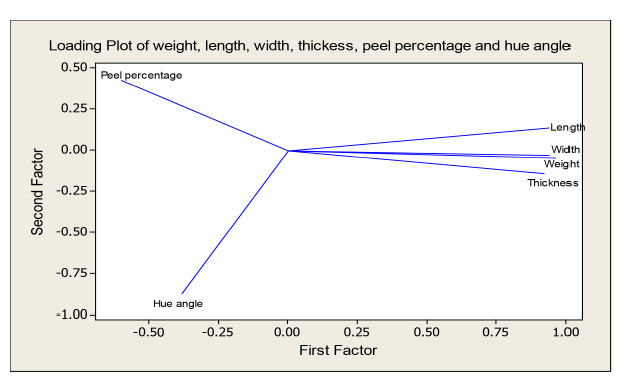
Loading plot of Principal Component 1 and 2 of the studied *50 Harumanis* accessions according to the six studied morphological traits (weight, length, width, thickness, peel percentage and hue angle).

Loading plot indicated in Fig. 3 presents the coefficients of each variable for the first PC versus the coefficients for the second PC. The first PC in the horizontal direction presented by four morphological traits namely fruit weight, length, thickness and width, while the second PC summarised the two variables of peel percentage and hue angle. The loading plot of the first PC consists of morphological variables like fruit weight, length, width and thickness are close to 1.00. Meanwhile, in the second factor, peel percentage loading value is close to 0.5 and hue angle loading is close to −1.00. The loading plot able to illustrate the strong influence of variables towards the PC except for peel percentage as this trait showed the least influence towards the PC.

**Figure 4:**
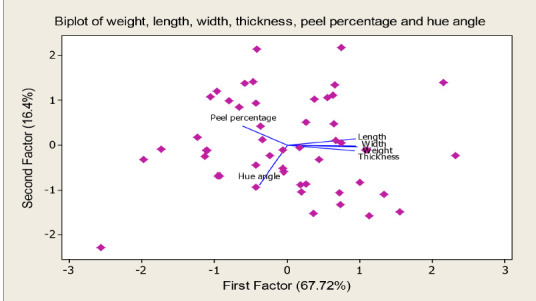
Biplot of Principal Component 1 and 2 of the studied *50 Harumanis* accessions according to the six studied morphological traits (weight, length, width, thickness, peel percentage and hue angle).

**Figure 5:**
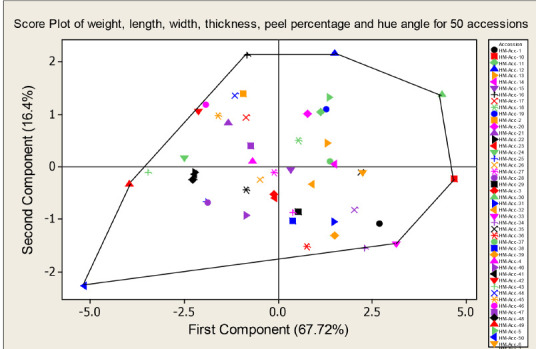
Two-dimensional score plot for the studied 50 *Harumanis* accessions of six components of the studied morphological traits (weight, length, width, thickness, peel percentage and hue angle).

A PC biplot constructed shows the super imposed of the variables on the plot as vectors. This biplot graph (Fig. 4) was comprised of the loading (Fig. 3) and score plot (Fig. 5). It was used to compare the different accessions diversity (Habibpour *et al.* 2012). According to the graphs (Figs. 4 and 5), *Harumanis* accessions such as HM-Acc 10, HM-Acc 12, HM-Acc 16, HM-Acc 30 and HM-Acc 50 were positioned at the vertex of polygon and are the farthest from the vertex point of origin (Figs. 4 and 5), hence they are more diversified than others. Meanwhile, HM-Acc 4, HM-Acc 15, HM-Acc 27 and HM-Acc 27 were positioned very near to each other and very close to the point origin, hence they are suggested to be less diversified.

### Clustering analysis

**Figure 6:**
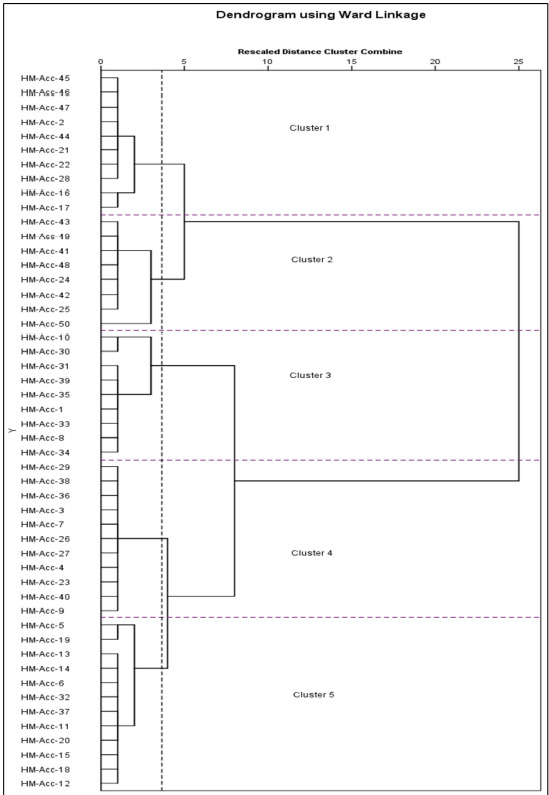
Final dendrogram showing five clusters as a result of cluster analysis (Ward method, squared Euclidean distances, *Z*-score standardisation of variables) using five morphological key descriptors on 50 local *Harumanis* samples. The cutting line for the cluster formation is marked as a dotted line.

The clustering analysis was characterised using fruit weight, thickness, width, length, peel percentage and hue angle as shown in Fig. 6 and Table 9. Ward’s dendrogram reflects the similarities and differences between *Harumanis* accessions according to the selected morphological characteristics which revealed maximum proportion of variation among the accessions. The 50 *Harumanis* accessions were mainly divided at the third node into five clusters with 10 (cluster 1), 8 (cluster 2), 9 (cluster 3), 11 (cluster 4) and 12 (cluster 5) accessions in different groups (Table 9 and Fig. 6). It was observed that the 50 *Harumanis* accessions were highly distributed into particular groups with no apparent location division. This can be clearly observed as multiple accessions were collected from different location such as HM-Acc 3 (Chelong Balik Bukit), HM-Acc 23 (Alor Ara Timur), and HM-Acc 40 (Simpang Empat) which were clustered together in the same group (cluster 4). Three clusters (cluster 1, 2 and 3) were comprised of fruits with similar fruit size of small and medium sizes. These clusters were differ distinctively in the flesh colour and peel percentage. Cluster 2 and cluster 4 fruit accessions reveal same two colour sets of fruit flesh (orange-yellow and yellow-orange), while cluster 1 was represented by one colour set of flesh color (orange-yellow). For peel percentage, highest peel percentage (14% to 18%) was observed in cluster 1 accession, whereas fruits in cluster 2 and 4 are having medium peel percentage value (cluster 2: 13% to 16%; cluster 4: 12% to 15%). Among five clusters, cluster 3 indicates *Harumanis* accessions with large size (> 500g), lowest peel percentage range (11% to 14%) and flesh with two different set of colours (orange and yellow-orange). Accessions in cluster 5 presented medium fruit size with the most diverse range value of peel percentage which is in between the minimum peel percentage till the maximum peel percentage (10% to 18%).

## DISCUSSION

Fruit characteristics were identified to have the strongest discriminating power in the previous studies and are useful for identifying particular landraces or morphological types (Sennhenn *et al.* 2014). CV among the *Harumanis* for several morphological traits such as fruit dimensional characteristics and flesh firmness showed high dispersion of the mean values and thus indicated high variability between the individuals (Table 2). The phenotypic diversity may governed by geographical location of the accessions as reported by previous finding of *Ziziphus* fruits (Markovski & Velkoska-Markovska 2015). Such findings are in line with the previous reports as indicated by Begum *et al*. (2013, 2014).

The results of morphological traits like dimensional characteristics, peel percentage and hue angle variations were robustly linked at the margin of statistical significance (*p* < 0.05) to the different locations and tree age as indicated in (Tables 3, 4, 5 and 6; Figs. 1 and 2). Further, the result is explained by employing the ANOVA in combination with Tukey post hoc test (Tables 3 and 4) and Kruskal-Wallis in combination with Dunn’s pairwise tests (Tables 5 and 6; Figs. 1 and 2) to point out the existence of morphological traits divergence across the two categories. These findings revealed the possible influence of distinct environmental factors such as human intervention (Singh *et al*. 2011), pollinators (Sánchez-Lafuente *et al*. 2005) and climate (Pregitzer *et al*. 2013), while the different age categories may associated with different level of constituents such as canopy size, number of leaves, respiration rates, enzymatic activities, antioxidant activities, organic acids contents and mineral contents (Meena & Asrey 2018). This finding is supported by an earlier study of environment interactions of plant and soil which reported the significance of environmental factors towards the mediation of plant morphological characteristics (Pregitzer *et al.* 2013). The environmental factors which attributed by the different locations and tree ages (plant structure and function) are inter-correlated. The factors such as light intensity, temperature, carbon availability, water availability influenced the plant system and further affecting the fruit growth specifically on its productivity (Singh *et al.* 2014).

Taken together, the result showed that *Harumanis* accessions from Simpang Empat acquired the smallest fruit weight and size when compared to the *Harumanis* accessions from Chelong Balik Bukit, Paya Kelubi and Santan with an exception of *Harumanis* Alor Ara Timur as the post hoc comparisons failed to give a significant result (*p* < 0.05) for any dimensional characteristic between these two groups. *Harumanis* Chelong Balik Bukit, *Harumanis* Paya Kelubi and *Harumanis* Santan attained bigger size and fruit weight whilst, *Harumanis* Alor Ara Timur was classified as medium fruit size. The data of present study shows that *Harumanis* fruits of older trees (33 to 35 years old) were relatively bigger compared to fruits of young (5 to 10 years old) and middle-aged (15 to 20 years old) trees as indicated in Table 6. *Harumanis* accessions collected from Simpang Empat and Alor Ara Timur are having smaller sizes as trees from both areas are categorised as young (5 to 10 years old) and middle-aged (15 to 20 years old). One of the possible factor is the larger canopy acquired by the older trees which lead to the optimum level of leaf-to-fruit ratio which enable the optimum uptake of the carbon sources, which further enable fruits to reach their optimum fruit size (Chacko *et al*. 1982; Reddy & Singh 1991). Contradictorily, young trees usually have smaller canopy, lesser number of leaves and all of these factors contributed to the lower photosynthesis rates in youth unexpanded leaves to obtain enough carbon sources and contribute to smaller size of fruits (Nakorn & Chalumpak 2016).

Other than that, previously it was stated that fruit sizes appeared to be controlled by multiple genes without complete dominance. At the same time, it is also concluded that fruit sizes were probably an expression of additive gene effects. This inheritance factor can be one of important potential factors contributing to the variation existence in *Harumanis* fruits sizes as indicated by descriptive statistic result for fruit weight which presented a high CVs of 20.69% which was greater than 20%. The value suggesting a great extent of fruit size variation as this trait was strong positively correlated with the fruit dimensional characteristics such as length, width and thickness (Table 2). The inheritance factor contribution was strongly supported by the previous findings as it was noticed that high degree of genotypic coefficient variation (GCV) along with phenotypic coefficient variation in the traits like fruit weight, fruit volume, pulp: stone ratio and total carotenoids. Hence, indicated high heritability along with high genetic advance estimated for the fruit weigh and fruit volume. A study on genotypic and phenotypic variability in mango indicated high value of fruit weight (85.20%) and thickness (83.12%) heritability. The higher heritability indicated that either these characters were simply inherited characters governed by a few major genes or it is an additive gene effect. If this trait confirmed to be under polygenic control, selection of this trait will be effective for crop improvement (Majumder *et al*. 2012).

In the correlation coefficient analyses, only a few parameters are considered due to the submission towards the Spearman’s assumption. From the findings, fruit dimensional characteristics, peel percentage and hue angle were selected as the most valuable key descriptors for the analyses due to the existence of significant differences across both categories (location and tree age) in which there are high correlation between the parameters and the variation composition of the *Harumanis* morphological traits can be explained. The fruit weight shows a strong significant positive linear correlation with fruit length, width and thickness. Meanwhile, brix values show negative correlation with hue angle. These findings are consistent with the previous results in which a strong positive correlation coefficient was also obtained between those traits in three species of *Ziziphus* genus (*Ziziphus nummularia*, *Ziziphus spina-christi* and *Ziziphus oxyphylla*) (Norouzi *et al*. 2017), sour cherry (*Prunus cerasus*) (Ganopoulos *et al*. 2016) and *Elaeagnus angustifolia* (Khadivi 2018). These significant correlations observed between phenotypic traits which contribute to fruit yield and quality will be so beneficial for any future plans of breeding programme (Ganopoulos *et al.* 2016. It also able to equip information on valuable traits in the evaluation of any accessions (Khadivi 2018).

According to Meena and Asrey (2018), higher respiration rate shown by fruits in older trees may attribute to higher peel thickness and peel percentage as both parameters have same palatability effects. The report opposed the findings of this study as the fruits of middle-aged (15 to 20 years old) and relatively old (33 to 35 years old) *Harumanis* trees attained lower peel percentage content compared to the peel percentage of young trees (5 to 10 years old) trees which attained the highest peel percentage. This result can be well analysed by performing the bivariate Spearman’s correlation coefficient, as it clearly able to explain the parameters correlation as the result showed a strong negative linear correlation between the peel percentage with weight (*r_s_* = −0.468), length (*r_s_* = −0.567), width (*r_s_* = −0.519) and thickness (*r_s_* = −0.519) (Table 7). These can be observed in the results of *Harumanis* fruits from orchard in Simpang Empat as it attained the lowest weight, thickness and highest peel percentage despite its young (5 to 10 years old) tree ages as presented by Tables 3, 4, 5 and 6. Other environmental factor such as human intervention which associated with the farming management system may attribute to these opposing results. Earlier, such finding was also reported on ‘Amrapali’ mango and one of the main factors identified is the lower level of potassium (K) in fruits from young trees which in turn disturbed the cell arrangement and resulted in higher peel thickness or peel percentage is mainly influenced by cell number, cell size and their arrangement in fruit tissue (Meena & Asrey 2018). Thus, a deep study dealing with some important chemical and biochemical aspects during both fruit development and ripening is needed.

The negative correlation (*r_s_* = −0.300, *p* < 0.05) in Table 7 between fruit brix value and hue angle able to point out a non-linear relationship between these two variables. The fruit brix value will increase as the hue angle value decreases, in which brix value contributes to the sweet taste of the fruit and the lower value of hue angle may prone to orange colour region. A few previous works have addressed the non-linear behaviour of these two parameters especially on the climacteric fruits (Jha *et al*. 2010; Soltani *et al*. 2010). The findings were in line with previous study in which reported by Khan *et al*. (2015) as the mango pulp usually has a sweet taste and as soon as the fruit ripens the colour of pulp changes from yellow to orange. Earlier, it was stated that the regulation of these two parameters were associated with ethylene production especially for mango, as it was identified as climacteric fruit species with a well-characterised peak in ethylene production and respiratory activity at the onset of ripening (Seymour *et al*. 1993; Tucker 1993; Adams-Phillips *et al*. 2004; Zaharah *et al*. 2011). This burst in ethylene production stems from an up-regulation of ethylene biosynthesis genes at the onset of ripening, resulting in autocatalytic ethylene production and an up-regulation of the components of ethylene perception and signalling (Klee & Clark 2004). These changes act as a key signal for the initiation and coordination of ripening in all climacteric fruits, and have been shown to regulate key genes that control colour change, fruit softening, cell wall breakdown, pathogen defence, and nutrient composition (Hobson & Grierson 1993; Tucker 1993; Alexander & Grierson 2002; Giovannoni 2004). These findings were supported by other study which stated that any biochemical and physiological changes during fruit ripening are literally driven by the coordinated expression of fruit ripening-related genes. These genes encode enzymes that participate directly in biochemical and physiological processes. It is also stated that the ethylene production within the fruit activates many other enzymes resulting in physiological changes such as the change of colour from green to red and the softening of the fruit (Hossain *et al*. 2014). Surprisingly, it has been reported that the absence or presence of a respiratory climacteric on the creeper depends upon prevailing environmental conditions (Bower *et al*. 2002). This observation is correlate with another finding which showed that the respiratory climacteric is most likely not an absolute activator of the ripening process, but secondary and resultant to the ripening procedure (Kaushlendra *et al*. 2016). However, the result of brix value was not statistically significant across the locations and tree age with a *p*-value more than 0.05. The results suggested insufficient evidence to reject the hypothesis which revealed no significant variation of the parameters across both the location and tree age. Thus, these findings explained that there are no influence of environmental factor and tree age towards the *Harumanis* brix values.

However, flesh colour showed different result as the hue angle were robustly linked at the margin of statistical significance (*p* < 0.05) to the different locations and tree age. Thus, the result suggested the influence of environmental factor and different tree developmental stages to the flesh colour variation. Earlier, light has been identified as one of the important elements which able to regulate carotenoid metabolism in plants (Zhang *et al*. 2015). For example, ripe ‘Keitt’ mango from Bahia, Brazil (hot climate) is reported to possess more than twice the β-carotene content than those from São Paulo (moderate climate) (Mercadante & Rodriguez-Amaya 1998). β-carotene has been emphasised as the main component that is interrelated with flesh colour and resulting in vitamin A values (Golob *et al*. 2012). Practically, light intensity was correlated with temperature, particularly in the open-air condition (He *et al*. 2018) and it was also demonstrated that the temperature was significantly linear to the light intensity (Gou *et al*. 2015). The carotenoid accumulation is in parallel with the intensity of the colour which is reflected by the hue angle, *h* value. In this study, *Harumanis* accessions from Chelong Balik Bukit and Paya Kelubi which cover the Chuping area are having the densest flesh colour (orange and orange-yellow) with a mean rank score of 22.20 and 8.40 for Dunn’s pairwise test respectively. Previous study on surface meteorological temperature observation data (January till June 2017) obtained from the Malaysian Meteorological Department involving two regions which are Chuping (representing orchards in Chelong Balik Bukit, Paya Kelubi, Santan and Alor Ara Timur) and Kangar (representing orchards in Simpang Empat) shows a consistent and only a slight difference in the temperature between the two regions with an average temperature of 28.4°C for Kangar and 27.7°C for Chuping (Yusuf *et al*. 2018b). Thus, it can be postulated that the temperature of Perlis region is not a crucial environmental factor in the flesh colour of *Harumanis* fruits (Yusuf *et al*. 2018b). Thus, other than environmental factor, tree age may possibly influence the carotenoid accumulation.

According to the result, the highest carotenoid accumulation explained by the lowest *h* value are strongly exhibited by the *Harumanis* fruits of relatively old trees (33 to 35 years old) from Chelong Balik Bukit, Paya Kelubi and Alor Ara Timur orchards compared to fruits of young trees (5 to 10 years old from Alor Ara Timur and Simpang Empat orchards) and middle-aged trees (15 to 20 years old from Santan orchards). This finding is in line with a previous study which reported that the highest carotenoids content was recorded in the pulp of fruits harvested from old trees (30 years old) (Meena & Asrey 2018). The linear relationship between carotenoid content and flesh colour is also reported in few previous studies. The fruit pulp was prominently orange in such early varieties of Mankurad and Furtado mangoes, mid-late variety of -RC-MS-1 (Bemcorado selection) mango, and also in late variety of Bardez Mussarat mango, which indicated the source for higher carotenoids contents (Desai & Dhandar 2000). The vegetative phase which is predominant in the early age of the plant usually resulted in higher sodium (N) content and lowered carotenoids synthesis and thus giving negative association with colour development in mango (Oosthuyse 1993; Martinez *et al*. 1997). Higher accumulation of calcium (Ca) was discovered in the fruits of 6 years old tree and might have further reduced the senescence process and thereby slowing the rate of chlorophyll breakdown and carotenoids synthesis in young tree fruits (Tingwa & Young 1974; Tirmazi & Wills 1981). However, the research on mango morphological traits performances according to the different tree age and environmental factor is still inadequate. Thus, further analyses on the differences *Harumanis* performances of morphological traits from different tree ages and the impact of specific environmental influence towards the traits performance would be a good approach to understand the underlying mechanism of the significant differences between those parameters stated. Other than that, it would be better if the colour trait performance of the accessions is assessed individually because the existence of morphological significant different results also may be governed by the dominants genes or number of genes. It is because of, though there was some dominance of light yellow colour to orange, the gene action was primarily additive both within and among loci for pulp colour (Iyer 1991). A few previous researches on the association of gene with the traits already performed and the findings showed significant association of gene loci with six traits of mango such as bloom, pulp colour, branch habit, ground skin colour, blush intensity and beak shape (Bally & Dillon 2018).

Further, those variables have been reduced by performing PCA analysis to reveal the general differences between the *Harumanis* accessions as numerical values and able to explain the variation among the individuals (Balkaya *et al*. 2010). The number of factors to be retained was determined according to the eigenvalues (Das *et al*. 2014). Therefore, in this analysis the first factor retains the information contained in 4.0638 of the original variables as given in Table 8. In this study, traits with high coefficients in the first and second (PCs) which are fruit weight, length, width, length, peel percentage and hue angle were considered important since these axes explain the biggest share of the total variation of 84.09% as indicated in Table 8. Though clear guidelines do not exist to determine the significance of a character coefficient, one rule of thumb is to treat coefficients > 0.6 as having a large enough effect to be considered as important (Jeffers 1967). The result is sufficient (84.1%) as it met a few of previous findings of PCA on mango which described the first ten components accounted for more than 86.07% of the total genetic variation in different mango varieties was accepted (Narvariya 2016) and the first six principal components axes took into account 75.12% of the total variance in the mango germplasm from the upper Athi river region of Eastern Kenya (Toili *et al*. 2016). In addition, the above-mentioned characters loaded high positively and negatively in the loading plots in Fig. 3, thus contributed more to the diversity and the selection of those characters is so effective as those traits able to differentiate the clusters greatly. In general, an identification of highly correlated morphological traits and the variation proportion contributed by selected parameters could be assessed by PCs analyses.

To better understand the overall diversity of the 50 *Harumanis* accessions, the data were analysed by cluster analysis that revealed the distribution of variety diversity and is displayed in Table 9 and Fig. 6. Categorising and characterising are necessary steps in the crop selection and breeding programmes (Markovski & Velkoska-Markovska 2015). The clustering analysis (Table 9 and Fig. 6) able to visualise the high distributional behaviour of the *Harumanis* accessions according to the selected morphological characteristics in the PCA analysis (weight, dimensional characteristic, peel percentage and hue angle). The clustering data shows that the *Harumanis* accessions were not grouped according to the location, opposing the previous reported by Sennhenn *et al.* in 2014. This finding was indicated by five clusters in Table 8 and Fig. 3, as multiple accessions collected from different locations were grouped in the same cluster such as Cluster 5, which consist of *Harumanis* accessions from Chelong Balik Bukit, Paya Kelubi, Alor Ara Timur and Santan. The 50 *Harumanis* accessions were effectively grouped together according to their close relationships associated with fruit morphological characteristics as indicated by the five clusters as shown in Table 9 and Figure 6. Overall, the clustering analysis results able to point out that the young trees are prone to produce fruits with small and medium sizes with a least dense flesh colour as the hue angle was quite high. These can be clearly seen in Cluster 2, as the cluster solely describe young tree performances. Meanwhile, middle-age and old trees mostly produced fruits with medium and large sizes with denser flesh colour as most of the accessions were grouped in the orange and orange-yellow colour sets.

Selection of morphological traits like fruit size and hue angle value would be effective for crop improvement study since they were possibly under polygenic control. However, previous study mentioned that the existence of variation in fruit weight under the same geographical region may be as a result of genotypic effect, cultivar and ecological condition (Markovski & Velkoska-Markovska 2015). In addition, since most of the plant characters of economic-important crops are governed by a group of genes and are highly influenced by environmental factor, hence it is difficult to judge whether the observed variability is heritable or due to environment. Thus, the optimisation of phenotypic variation into its heritable and non-heritable components for enhancement of genotypes collections is so important (Azam-Ali *et al*. 2006). Earlier, a study on the evaluation of the genetic variability for fruit quality in mango progenies showed the existence of genetic variability among progenies with differences in the progeny performance for the traits (Nayak *et al*. 2013).Thus, the individual analysis on the selected *Harumanis* which showed the desirable and economically traits would be a good resolution for future selection of potential parent and their subsequent prediction of progeny performance. Further, *Harumanis* crop breeding improvement can be assessed by selection of highly potential parent with highly commercial traits and comparing them against standard commercial varieties under replicate trials in combination with genetic study on the selected traits to confirm the distinctiveness and superiority. Therefore, the collection of desirable genotypes and a hybridisation programme may be initiated involving the evaluation of phenotypic diversity among genotypes as an important consideration for classification, utilisation of germplasm resources and breeding programme (Khadivi-Khub & Anjam, 2014).

## CONCLUSION

Broad phenotypic diversity was detected within this cultivar with high distribution exists within the intra-cultivar of 50 *Harumanis* accessions from different locations and tree ages. This study, in overall, shows that different location which associated with environmental factors and tree age have robust significance on *Harumanis* morphological fruits variation. For instance, this study found that *Harumanis* fruits of older trees (33 to 35 years old) were relatively bigger compare to the fruits of young (5 to 10 years old) and middle-aged (15 to 20 years old) trees, whilst for flesh colour, the most dense flesh colour was exhibited by the *Harumanis* accessions of Paya Kelubi. The data further suggest that morphological traits (weight, dimensional characteristics, peel percentage and hue angle) are useful to be utilised for *Harumanis* descriptors establishment as these parameters able to differentiate the *Harumanis* cultivar effectively up to 84.1% total variation accounted in the PCA analyses. The current findings hopefully can be employed as a fundamental basis for the advancement of *Harumanis* crop breeding programme.

## Figures and Tables

**Table 1:** List of *Harumanis* mango accessions and their collection sites. Location 1: Chelong Balik Bukit, Padang Besar (6.535464° [N],100.288332° [E]), Location 2: Paya Kelubi, Padang Besar (6.535175° [N], 100.253759° [E]), Location 3: Alor Ara Timur, Arau (6.456431° [N], 100.263158° [E]), Location 4: Santan, Kangar (6.482817° [N], 100.230928° [E]) and Location 5: Simpang Empat, Kangar (6.322332° [N], 100.206032° [E]).

No	Accession Name	Location	Age (Year)	No	Accession Name	Location	Age (Year)
S1	HM-Acc-1	1	35	S26	HM-Acc 26	3	9
S2	HM-Acc-2	1	35	S27	HM-Acc 27	3	9
S3	HM-Acc-3	1	35	S28	HM-Acc-28	3	33
S4	HM-Acc-4	1	35	S29	HM-Acc-29	3	33
S5	HM-Acc-5	1	35	S30	HM-Acc 30	3	33
S6	HM-Acc-6	1	35	S31	HM-Acc-31	4	20
S7	HM-Acc-7	1	35	S32	HM-Acc-32	4	20
S8	HM-Acc-8	1	35	S33	HM-Acc-33	4	20
S9	HM-Acc-9	1	35	S34	HM-Acc-34	4	20
S10	HM-Acc-10	1	35	S35	HM-Acc-35	4	20
S11	HM-Acc-11	2	33	S36	HM-Acc-36	4	20
S12	HM-Acc-12	2	33	S37	HM-Acc-37	4	20
S13	HM-Acc-13	2	33	S38	HM-Acc-38	4	20
S14	HM-Acc-14	2	33	S39	HM-Acc-39	4	20
S15	HM-Acc-15	2	33	S40	HM-Acc-40	4	15
S16	HM-Acc-16	2	33	S41	HM-Acc-41	5	10
S17	HM-Acc-17	2	33	S42	HM-Acc-42	5	10
S18	HM-Acc-18	2	33	S43	HM-Acc-43	5	10
S19	HM-Acc-19	2	33	S44	HM-Acc-44	5	10
S20	HM-Acc-20	2	33	S45	HM-Acc-45	5	10
S21	HM-Acc-21	3	5	S46	HM-Acc-46	5	10
S22	HM-Acc-22	3	5	S47	HM-Acc-47	5	10
S23	HM-Acc-23	3	5	S48	HM-Acc-48	5	10
S24	HM-Acc-24	3	9	S49	HM-Acc-49	5	10
S25	HM-Acc-25	3	9	S50	HM-Acc-50	5	10

**Table 2:** The min, max, mean, standard deviation and CV of traits in the studied individuals of *Harumanis*.

No	Character	Unit	Min	Max	Mean	SD	CV (%)
1	Weight	g	263.00	670.33	435.05	89.72	20.62
2	Length	cm	10.00	14.31	11.98	0.89	7.46
3	Width	cm	6.07	8.45	7.31	0.52	7.16
4	Thickness	cm	5.63	7.69	6.77	0.50	7.39
5	Peel firmness	(N/s)	3.054772	7.235673	5.335907	0.969012	18.16
6	Flesh firmness	(N/s)	0.701176	2.933823	1.384797	0.477329	34.47
7	TSS	(°)	11.76	18.23	16.08	1.00	6.22
8	Peel percentage	(%)	10	18	14	2.07	14.94
9	Pulp percentage	(%)	53	74	67	4.39	6.58
10	Stone percentage	(%)	9	19	12	1.76	14.23
11	Hue angle	(°)	68.09	87.13	76.29	3.29	4.31

**Table 3:** ANOVA for five morphological traits across different location.

		Sum of squares	df	Mean square	*F*	Sig.
Weight	Between Groups	144666.955	4	36166.739	6.516	0.000
	Within Groups	249779.733	45	5550.661		
	Total	394446.688	49			
Length	Between Groups	17.021	4	4.255	8.665	0.000
	Within Groups	22.099	45	0.491		
	Total	39.120	49			
Width	Between Groups	5.457	4	1.364	8.275	0.000
	Within Groups	7.418	45	0.165		
	Total	12.875	49			
Thickness	Between Groups	6.417	4	1.604	13.811	0.000
	Within Groups	5.227	45	0.116		
	Total	11.644	49			
Peel firmness	Between Groups	22.197	4	5.549	10.487	0.000
	Within Groups	23.813	45	0.529		
	Total	46.010	49			

**Table 4:** Post Hoc Tests Multiple Comparisons, Tukey HSD, on the fruit dimensional characteristics across five location (the mean difference is significant at the 0.05 level).

Dependent variable	(I) LOCATION	(J) LOCATION	Mean Difference (I–J)	Std. Error	Sig.	95% Confidence Interval	

						Lower Bound	Upper Bound
Weight	Chelong Balik Bukit	Simpang Empat	140.41567^*^	33.31865	0.001	45.7424	235.0889
	Paya Kelubi	Simpang Empat	105.53667^*^	33.31865	0.022	10.8634	200.2099
	Santan	Simpang Empat	151.56667^*^	33.31865	0.000	56.8934	246.2399
	Simpang Empat	Chelong Balik Bukit	−140.41567^*^	33.31865	0.001	−235.0889	−45.7424
	Simpang Empat	Paya Kelubi	−105.53667^*^	33.31865	0.022	−200.2099	−10.8634
	Simpang Empat	Santan	−151.56667^*^	33.31865	0.000	−246.2399	−56.8934
Length	Chelong Balik Bukit	Simpang Empat	1.38196^*^	0.31340	0.001	0.4915	2.2725
	Paya Kelubi	Alor Ara Timur	0.92773^*^	0.31340	0.037	0.0372	1.8182
	Paya Kelubi	Simpang Empat	1.46996^*^	0.31340	0.000	0.5795	2.3605
	Alor Ara Timur	Paya Kelubi	−0.92773^*^	0.31340	0.037	−1.8182	−.0372
	Santan	Simpang Empat	1.36996^*^	0.31340	0.001	0.4795	2.2605
	Simpang Empat	Chelong Balik Bukit	−1.38196^*^	0.31340	0.001	−2.2725	−0.4915
	Simpang Empat	Paya Kelubi	−1.46996^*^	0.31340	0.000	−2.3605	−0.5795
	Simpang Empat	Santan	−1.36996^*^	0.31340	0.001	−2.2605	−0.4795
Width	Chelong Balik Bukit	Simpang Empat	0.79241^*^	0.18158	0.001	0.2765	1.3084
	Paya Kelubi	Simpang Empat	0.66441^*^	0.18158	0.006	0.1485	1.1804
	Santan	Simpang Empat	0.95541^*^	0.18158	0.000	0.4395	1.4714
	Simpang Empat	Chelong Balik Bukit	−0.79241^*^	0.18158	0.001	−1.3084	−0.2765
	Simpang Empat	Paya Kelubi	−0.66441^*^	0.18158	0.006	−1.1804	−0.1485
	Simpang Empat	Santan	−0.95541^*^	0.18158	0.000	−1.4714	−0.4395
Thickness	Chelong Balik Bukit	Simpang Empat	0.90537^*^	0.15242	0.000	0.4723	1.3384
	Paya Kelubi	Simpang Empat	0.61037^*^	0.15242	0.002	0.1773	1.0434
	Alor Ara Timur	Santan	−0.48800^*^	0.15242	0.020	−0.9211	−0.0549
	Alor Ara Timur	Simpang Empat	0.54437^*^	0.15242	0.007	0.1113	0.9774
	Santan	Alor Ara Timur	0.48800^*^	0.15242	0.020	0.0549	0.9211
	Santan	Simpang Empat	1.03237^*^	0.15242	0.000	0.5993	1.4654
	Santan	Chelong Balik Bukit	−0.90537^*^	0.15242	0.000	−1.3384	−0.4723
	Santan	Paya Kelubi	−0.61037^*^	0.15242	0.002	−1.0434	−0.1773
	Simpang Empat	Alor Ara Timur	−0.54437^*^	0.15242	0.007	−.9774	−0.1113
	Simpang Empat	Santan	−1.03237^*^	0.15242	0.000	−1.4654	−0.5993

**Table 5:** Results on Kruskal-Wallis Rank test for hue angle and peel percentage across five locations.

		Hue angle			Peel percentage		

Place	N	Median	Ave Rank	Z	Median	Ave Rank	Z
Alor Ara Timur	10	77.99	31.4	1.44	14.50	30.6	1.24
Chelong Balik Bukit	10	76.06	22.2	−0.80	12.00	18.5	−1.70
Paya Kelubi	10	73.44	8.4	−4.15	13.50	22.9	−0.63
Santan	10	79.00	37.0	2.79	12.50	16.6	−2.17
Simpang Empat	10	77.25	28.4	0.72	15.50	39.0	3.26
Overall	10		25.5			25.5	
		H = 16.13 DF = 4 P = 0.003			H = 15.96 DF = 2 P = 0.000		
		H = 16.54 DF = 4 P = 0.002			H = 15.96 DF = 2 P = 0.000		
		(adjusted for ties)			(adjusted for ties)		

**Table 6:** Results on Kruskal-Wallis Rank test for seven morphological traits across tree age categories.

		Weight			Length			Width			Thickness		

Age	N	Median	Ave Rank	Z	Median	Ave Rank	Z	Median	Ave Rank	Z	Median	Ave Rank	Z
Middle	10	495.6	36.2	1.44	12.35	32.9	1.78	7.635	38.4	3.12	7.215	39.2	3.32
Old	23	451.8	30.0	−0.80	12.41	32.4	3.10	7.460	29.8	1.92	6.950	29.8	1.91
Young	17	361.5	13.2	−4.15	11.25	11.8	−4.77	7.050	12.1	−4.65	6.380	11.7	−4.81
Overall	50		25.5			25.5			25.5			25.5	
		H = 19.69 DF = 2 P = 0.000			H = 22.78 DF = 2 P = 0.000			H = 24.02 DF = 2 P = 0.000			H = 26.08 DF = 2 P = 0.000		
					H = 22.78 DF = 2 P = 0.000			H = 24.03 DF = 2 P = 0.000			H = 26.09 DF = 2 P = 0.000		
					(adjusted for ties)			(adjusted for ties)			(adjusted for ties)		

		Peel firmness			Peel percentage			Hue angle		

Age	N	Median	Ave Rank	Z	Median	Ave Rank	Z	Median	Ave Rank	Z
Middle	10	4.995	20.5	−1.21	12.50	16.6	−2.17	79.00	37.0	2.79
Old	23	4.916	19.0	−2.93	13.00	20.6	−2.18	74.50	17.0	−3.82
Young	17	6.420	37.3	4.11	15.00	37.4	4.13	77.32	30.3	1.67
Overall	50		25.5			25.5			25.5	
		H = 16.94 DF = 2 P = 0.000			H = 17.58 DF = 2 P = 0.000			H = 15.96 DF = 2 P = 0.000		
					H = 18.02 DF = 2 P = 0.000			H = 15.96 DF = 2 P = 0.000		
					(adjusted for ties)			(adjusted for ties)		

**Table 7:** Correlation coefficient of all *Harumanis* fruit morphological characteristics pairs.

Character		Weight	Length	Width	Thickness	Peel firmness	Flesh firmness	TSS	Peel %	Pulp %	Stone %	Hue angle
Weight	*r_s_*	1										
Length	*r_s_*	0.915**	1									
Width	*r_s_*	0.918**	0.828**	1								
Thickness	*r_s_*	0.887**	0.771**	0.889**	1							
Peel firmness	*r_s_*	−0.437**	−0.485**	−0.358*	−0.460**	1						
Flesh firmness	*r_s_*	0.321*	0.322*	0.345*	0.420**	−0.246	1					
TSS	*r_s_*	0.182	0.222	0.215	0.077	−0.058	0.074	1				
Peel %	*r_s_*	−0.567**	−0.519**	−0.519**	−0.581**	0.542**	−0.426**	−0.045	1			
Pulp %	*r_s_*	−0.057	−0.178	−0.064	−0.055	0.098	0.079	−0.004	−0.195	1		
Stone %	*r_s_*	−0.572**	−0.606**	−0.579**	−0.604**	0.281*	−0.271	−0.011	0.322*	0.043	1	
Hue angle	*r_s_*	−0.153	−0.354*	−0.092	−0.029	0.203	−0.096	−0.300*	0.000	0.203	0.268	1

*Notes*: For explanation of character symbols, see Table 7;

* Correlation is significant at the 0.05 level.;

** Correlation is significant at the 0.01 level

**Table 8:** Eigenvalue, percentage variance and cumulative variance for 50 *Harumanis* accessions based on six morphological traits.

	PC1	PC2	PC3	PC4	PC5	PC6
Eigenvalue	4.0638	0.9822	0.6390	0.1900	0.0772	0.0479
% total variance	0.677	0.164	0.106	0.032	0.013	0.008
% cumulative	0.677	0.841	0.947	0.979	0.992	1.000
Traits	PC1	PC2	PC3	PC4	PC5	PC6
Weight	0.478	−0.047	0.165	−0.328	0.002	0.796
Length	0.467	0.136	0.028	−0.643	−0.236	−0.542
Width	0.468	−0.030	0.279	0.263	0.761	−0.234
Thickness	0.459	−0.143	0.215	0.599	−0.598	−0.080
Peel percentage	−0.298	0.429	0.846	−0.060	−0.088	0.004
Hue angle	−0.189	−0.880	0.364	−0.218	−0.008	−0.103

**Table 9:** Minimum and maximum value of five quantitative and one qualitative key descriptors used for morphological classification of 50 *Harumanis* mango samples separately for the five identified clusters.

Classification	Cluster 1 (*n* = 10)		Cluster 2 (*n* = 8)		Cluster 3 (*n* = 9)		Cluster 4 (*n* = 11)		Cluster 5 (*n* = 12)	
Accession (HMAcc-)	2, 16, 17, 21, 22, 28, 44, 45, 46 and 47		24, 25, 41, 42, 43, 48, 49 & 50		1, 8, 10, 30, 31, 33, 34, 35 & 39		3, 4, 7, 9, 23, 26, 27, 29, 38, 36 & 40		5, 6, 9, 11, 12, 13, 14, 15, 18, 20, 32 and 37	
Location	2, 3, and 5		3 and 5		1 and 4		1, 3, and 4		1, 2, 3, and 4	
Age (year)	5, 10, 33, and 35 (young and old tree)		9 and 10 (young tree)		20, 33, and 35 (middle-aged and old tree)		5, 9, 15, 20, 33, and 35 (young, middle-aged and old tree)		20, 33, and 35 (middle-aged and old tree)	

Morphological characteristics										

Quantitative										

Fruit weight (g)	329.33–424.83	Small and medium size	263.00–402.00	Small and medium size	513.00–670.33	Large size	341.33–460.17	Small and medium size	419.33–530.67	Medium size
Fruit length (cm)	10.61–12.16		9.89–11.37		12.39–14.31		10.67–12.06		11.91–13.25	
Fruit width (cm)	6.96–7.29		6.07–6.75		7.69–8.45		6.87–7.57		7.26–7.82	
Fruit thickness (cm)	6.00–6.79		5.63–6.34		7.15–7.69		6.41–7.16		6.62–7.26	
Peel percentage (%)	14–18		13–16		11–14		12–15		10–18	
Qualititative										
Hue angle (°)	70.12–79.32	Orange-yellow group	73.99–87.13	Orange-yellow and yellow-orange group	68.38–81.09	Orange and yellow-orange group	75.92–80.11	Orange-yellow and yellow-orange group	68.09–76.83	Orange and orange-yellow group
